# Genetic differentiation at probe SNPs leads to spurious results in meQTL discovery

**DOI:** 10.1038/s42003-023-05658-5

**Published:** 2023-12-21

**Authors:** Gillian L. Meeks, Brenna M. Henn, Shyamalika Gopalan

**Affiliations:** 1 Graduate Program in Integrative Genetics and Genomics, University of California Davis, Davis, CA 95616 USA; 2 Department of Anthropology, University of California Davis, Davis, CA 95616 USA; 3 UC Davis Genome Center, University of California Davis, Davis, CA 95616 USA; 4 Department of Evolutionary Anthropology, Duke University, Durham, NC 27708 USA

**Keywords:** DNA methylation, Methylation analysis, Genetic variation, Quantitative trait loci, Epigenomics

## Introduction

**arising from** B. Li et al. *Communications Biology* 10.1038/s42003-022-03353-5 (2022)

DNA methylation variation broadly mirrors genetic variation, capturing population-level patterns that largely reflect global geography^1–4^; however, little is known about how genetic admixture shapes these relationships (but see refs. ^5,6^). To address this gap, Li et al. analyzed DNA methylation variation in African-Americans and found that, by incorporating local genetic ancestry (LA) information, they could identify novel ancestry-specific genetic effects on DNA methylation. However, our re-analysis finds that a significant proportion of their results involve methylated sites whose assay probe sequence contains at least one genetic variant that is strongly differentiated between European- and African-ancestry populations. Therefore, we hypothesize that many of the ancestry-specific signals reported by Li et al. actually arise from differential hybridization efficiency^7,8^, creating technical artifacts whose effects are confounded with LA.

**Fig. 1:**
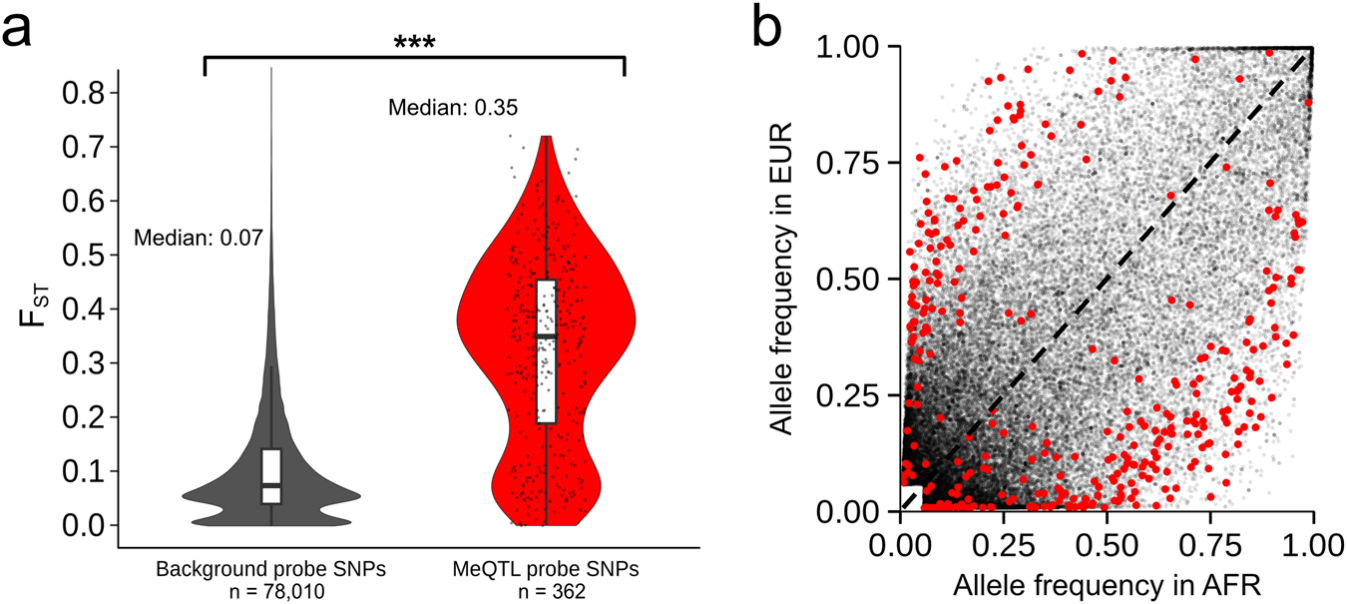
SNPs in Li et al.’s meQTL CpG probes are more differentiated than SNPs in non-meQTL CpG probes between 1000 Genomes EUR and AFR super-populations. **a** *F*_st_ distributions between meQTL (red) and non-meQTL (gray) probe SNPs are significantly different (two-tailed *t*-test *p*-value = 2.2e−16, 95% confidence interval for the difference in mean *F*_st_ [0.20–0.23]). The boxplots show the median (horizontal line), interquartile range (box), and 1.5x interquartile range (whiskers). **b** Probe SNP frequencies in AFR vs. EUR of the same probe SNPs (red points, Li et al.’s meQTL; gray points, non-meQTL) further highlight that they tend to be significantly differentiated, i.e., they tend to deviate from the dashed black line that indicates identical EUR and AFR allele frequencies.

**Fig. 2:**
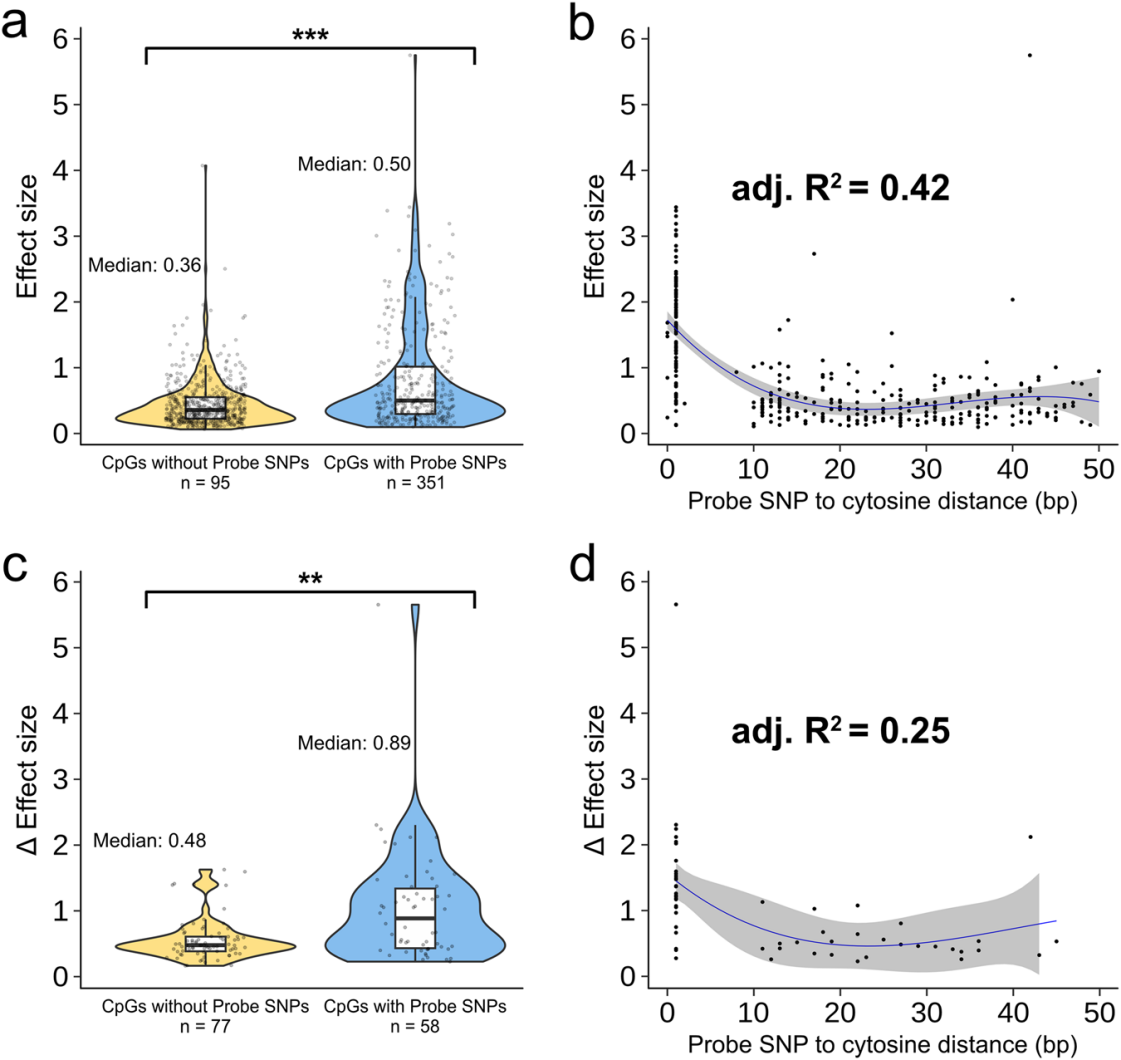
Probe SNPs bias effect size estimates. **a** MeQTL involving CpGs with probe SNPs (blue) have larger effect sizes than those without (yellow) (95% confidence interval for difference in mean effect size [0.28, 0.45], two-tailed *t*-test *p*-value = 1.44e−15). **b** A cubic regression of the distance between the probe SNP and target CpG explains 42% of the variation in meQTL effect size for the 351 CpG probes with SNPs (*F*-test *p*-value over a quadratic model = 0.0006). **c** LA-specific meQTL involving CpGs with probe SNPs (blue) have larger differences in effect sizes (Δ) between European and African local ancestry than those without (yellow) (95% confidence interval for difference in mean Δ effect size [0.24, 0.70], two-tailed *t*-test *p*-value = 0.0002). **d** A quadratic regression of the distance between the probe SNP and target CpG explains 25% of the variation in Δ effect sizes for the 58 CpG probes with SNPs (*F*-test *p*-value over a linear model = 0.014). The boxplots in **a** and **c** show the median (horizontal line), interquartile range (box), and 1.5× interquartile range (whiskers). The ribbons in **b** and **d** show 2 standard errors above and below the model predictions.

Li et al. identified 1284 cytosine-guanine dinucleotides (CpGs) where DNA methylation level is associated with LA. Noting the high heritability of DNA methylation at these CpGs (mean *h*^2^ of 45%), they then scanned for methylation quantitative trait loci (meQTL), looking within 1 Mb of each LA-associated CpG for single nucleotide polymorphisms (SNPs) associated with methylation level. Under a conventional meQTL model, assuming identical effect sizes regardless of LA context (i.e., an ‘LA-naïve’ approach), they identify 1269 independent meQTL associations involving 946 unique CpGs. When they allow meQTL effect sizes to vary across African and European haplotypes (i.e., an ‘LA-aware’ approach) they identify 1268 independent meQTL involving 135 unique CpGs, 152 of which showed significant effect size heterogeneity. However, through careful probe region designation using a custom software package^9^ and cross-referencing with a comprehensive SNP set, we found that some of the largest ancestry-specific effects identified by Li et al. involved CpG sites whose array probe sequence contains a common SNP. Standard quality control procedures recommend filtering out such CpGs from analysis because probe sequence mismatches can impact hybridization efficiency, affecting the reliability of the assay^8^. Furthermore, the probe SNPs we identify tend to be strongly differentiated between European and African-ancestry populations (Fig. 1), a pattern that we would expect to result in spurious LA-DNA methylation associations. Finally, we demonstrate that the probe SNP-CpG site distance in base pairs is strongly predictive of meQTL effect size or LA-specific difference (Δ) of effect size in the LA-naïve and LA-aware approaches, respectively (Fig. 2). This pattern would likewise be expected if associations were driven primarily by technical artifacts^7^. Given our findings, we argue that Li et al.’s conclusion of widespread LA-associated heterogeneity in meQTL effect sizes should be re-evaluated.

We cross-referenced the probe sequences of all CpGs analyzed by Li et al. with a comprehensive set of variants likely to be segregating in African-American populations (see the “Methods” section). We find that 37.5% of Li et al.’s meQTL-associated CpGs contain at least one common probe SNP, compared to only 16.1% of CpGs not associated with meQTL (non-meQTL CpGs). We then calculated Cockerham and Weir’s *F*_st_ between 1000 Genomes Phase 3^10^ African (AFR) and European (EUR) references for both meQTL and non-meQTL probe SNPs (Fig. 1). We find that meQTL probe SNPs are significantly more differentiated between African and European populations than non-meQTL probe SNPs (median *F*_st_ of 0.35 versus 0.07; Fig. 1). Therefore, if these meQTL-associated probe SNPs do impact hybridization efficiency, they would generate spurious associations between LA and DNA methylation. We find that for 10% (95/946) of CpGs identified by the LA-naïve approach, which affects 18% of all LA-naïve meQTL associations, a strongly differentiated (i.e. minimum *F*_st_ of 0.1; median *F*_st_ of 0.39) probe SNP directly impacts the presence or absence of the CpG site. This represents a lower bound on the proportion of Li et al.’s results that are impacted by technical artifacts, as individuals with the CpG loss variant would be incorrectly inferred to have a low level of DNA methylation. Of the CpGs identified in the set of meQTL showing significant effect size heterogeneity, this proportion is even higher; 22% (30/135) of CpGs, impacting 50% of these LA-specific meQTL associations (minimum *F*_st_ of 0.1; median *F*_st_ of 0.44).

We then assessed the relationship between meQTL effect size and CpG-probe SNP distance. Previous research has shown that the closer the probe SNP is to the target cytosine, the greater its impact on hybridization efficiency^7^. First, as expected, we found that meQTL with probe SNPs had significantly higher reported effect sizes (and Δ effect sizes) than those without (Fig. 2a, c). For the LA-naïve results, we found that a cubic regression best describes the relationship between effect size and CpG-SNP distance (Fig. 2b). Importantly, under this model, CpG-SNP distance alone explains 42% of the variance in effect size. For the LA-aware results, a quadratic regression gave the best fit, with CpG-SNP distance explaining 25% of the variance in Δ (Fig. 2d). This non-linear relationship is driven by the much larger relative effect sizes of SNPs at positions 0 and 1^8^ because these SNPs would conflate a loss-of-CpG genotype with a lack of methylation.

As epigenetics research considers a greater range of human diversity, assessing the influence of genetic ancestry on DNA methylation will become increasingly important. However, rigorous quality control measures must be taken to ensure that technical features of the array do not confound analyses. We have shown that commercial DNA methylation array probe sequences contain genetic variation that is common in non-European populations, specifically African and African-American populations. We argue that such CpGs should not be analyzed, per established quality control guidelines^8^. Rigorous probe exclusion is especially important in analyses of admixed populations, where multiple differentiated genetic ancestries are combined within the study cohort. Our analyses find that the LA-associated CpGs identified by Li et al. are enriched for probe SNPs that are strongly differentiated between European and African genetic ancestries. This likely generated spurious associations between DNA methylation levels and LA, driven by the fact that those AFR and EUR haplotypes either do not bind the CpG probe sequences on the array with equal efficiency, or they are differentiated with respect to the presence of the CpG. At a minimum, for 18% of meQTL identified via the LA-naïve approach and for 50% of meQTL identified as LA-specific, SNPs directly affect the presence of the CpG site. However, as previously noted, SNPs throughout the probe sequence can bias effect estimates^7,8^. This bias is evident in our analysis showing that the proximity of a probe SNP to the target cytosine alone explains 42% of the variance in Li et al.’s estimated meQTL effect sizes. While real biological effects also tend to be stronger with increased SNP-CpG proximity, a recent large-scale analysis of 27 million *cis*-meQTL found that on the order of 50 bp (i.e. the length of a probe sequence), meQTL–CpG distance explains <1% of the variation in effect size^11^. Therefore, technical biases, rather than real biological effects, are the most likely drivers of the pattern we observe.

In conclusion, we caution against correcting for local ancestry in future meQTL studies unless technical biases are rigorously accounted for. This must include cross-referencing methylation probe coordinates with a comprehensive set of common variants, such as the 1000 Genomes panel^10^. To this end, we present probeSNPffer^9^, a tool that performs this cross-referencing step (see the “Methods” section). We hypothesize that many of the variants in our list of reference SNPs were removed from consideration by Li et al.’s strict quality control criteria, but are nevertheless very likely to be present in their sample. For example, there are over 14 million variants segregating at >1% frequency in the 1000 Genomes ASW (*n* = 61), whereas Li et al. retained only 4.7 million variants at >1% frequency in their much larger sample (*n* = 1031). A large proportion of these nearly 10 million missing variants assuredly segregate in their sample and must be considered when filtering CpGs, especially when seeking to understand the impact of local ancestry on DNA methylation in admixed populations.

## Methods

We defined CpG probe regions as the 50 base pair (bp) sequence downstream or upstream of the target cytosine for forward and reverse strand targeted probes, respectively, along with the single base extension position for Type 1 probes and the C/T extension position for Type 2 probes. We cross-referenced these 51 bp probe regions with a set of SNPs exhibiting a minor allele frequency of at least 5% in either the 1000 Genomes Phase 3^10^ European (EUR) or African (AFR) super-population to identify a set of probe SNPs. To confirm that these SNPs are relevant for analyses of African Americans, we checked if these probe SNPs also segregate in the 1000 Genomes Southwest US African-Americans (ASW). Indeed, we found that 434 of the 439 probe SNPs are present at a frequency of at least 1% in this subset of AFR (*n* = 61). Only fluorescent color-channel switching SNPs (non-A/T or C/G SNPs) at the single base extension position for Type 1 probes were considered. We note that Li et al. did remove some CpG probes that contain SNPs close to the target cytosine in their quality control process, as we see a reduced number of probe SNPs within 10 bp of the target cytosine for their meQTL-associated CpGs (Supplementary Fig. 1). However, they appear to have missed 97 minus-strand targeted probes with a SNP at the *p* + 1 position (the C position of the minus genomic strand), compared to missing only 3 at the C position of forward-strand targeted probes. This was perhaps due to errors in defining the probe region (Supplementary Fig. 1, probe region diagram adapted from ref. ^12^).

### Reporting summary

Further information on research design is available in the Nature Portfolio Reporting Summary linked to this article.

### Supplementary information

Supplementary Information

Reporting Summary

## Data Availability

Data associated with Figs. 1 and 2 can be accessed via 10.5281/zenodo.10076886^13^. 1000 Genomes Phase 3^10^ data can be accessed via http://ftp.1000genomes.ebi.ac.uk/vol1/ftp/phase3/. The data from 27 million *cis*-meQTL^11^ are accessible via https://ftp.ncbi.nlm.nih.gov/eqtl/original_submissions/FHS_meQTLs/.

## Code availability

Our R package for identifying probe SNPs is available at https://github.com/gillianmeeks/probeSNPffer^9^.
