# Supplementary material for: Genetic differentiation at probe SNPs leads to spurious results in meQTL discovery
Source: Commun Biol. 2023 Dec 21;6:1295. doi: 10.1038/s42003-023-05658-5 (PMC10739831; doi:10.1038/s42003-023-05658-5)
Supplement: Supplementary file 1 — Supplementary Information [file 42003_2023_5658_MOESM1_ESM.pdf]

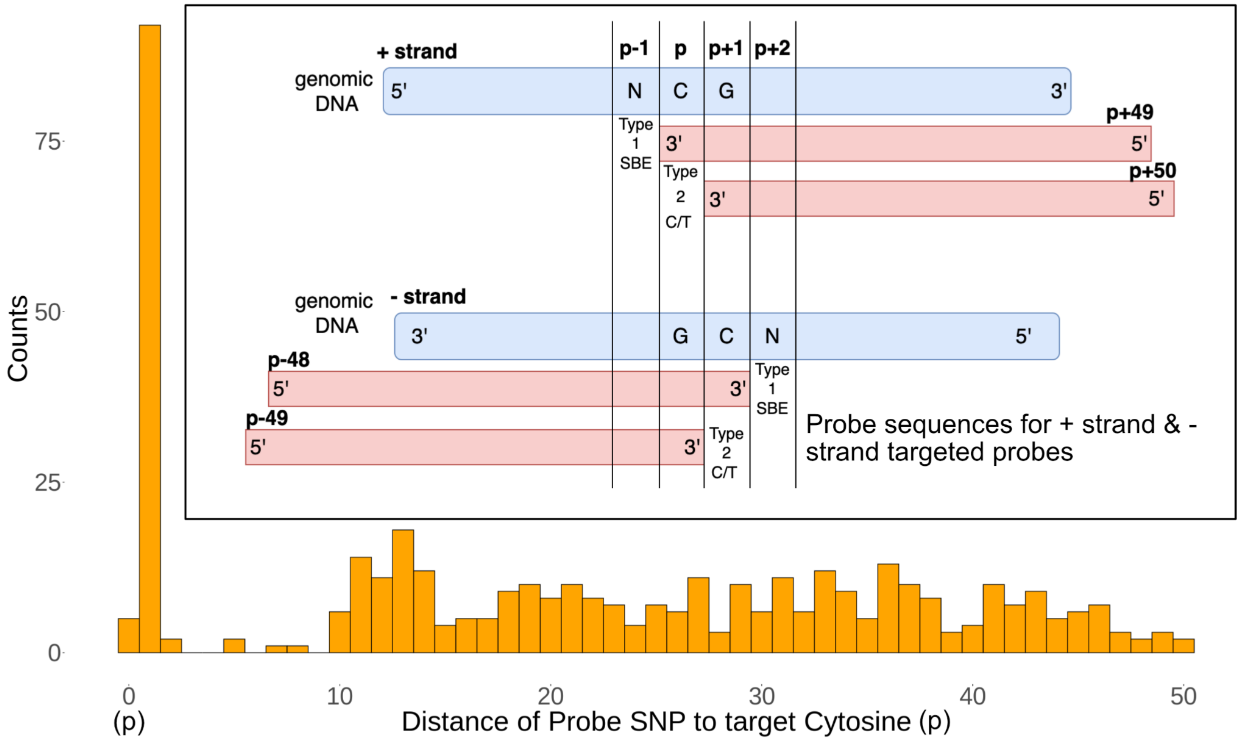

Supplementary Figure 1: The frequency of probe SNPs in Li et al. relative to the target cytosine position, defined as position 0 (p). The higher density of SNPs at the CpG site relative to the surrounding 50 bp sequences is likely due to hypermutability of CpGs. The formula for determining the coordinates of the probe sequence depends on target strand and probe type, and is shown in the inset (SBE stands for single base extension). Blue bars represent the forward (+) and reverse (-) genomic DNA strands, while the red bars represent the 50 bp sequence of Type 1 and Type 2 probes. The probe regions are marked with reference to the forward genomic strand coordinate of the target cytosine (position p) in the Illumina manifest, e.g. the probe region for Type 1, reverse (-) strand targeted probes extends from p-48 to p+2 to include the SBE position at p+2.
